# Antiphospholipid Syndrome and Antibodies

**DOI:** 10.1155/2014/942869

**Published:** 2014-08-18

**Authors:** Jozélio Freire de Carvalho, Roger Abramino Levy, Yehuda Shoenfeld

**Affiliations:** ^1^Rheumatology Division, Aliança Medical Center, 41810-080 Salvador, BA, Brazil; ^2^Rheumatology Division, Faculdade de Ciências Medicas, Universidade do Estado do Rio de Janeiro, 20550-900 Rio de Janeiro, RJ, Brazil; ^3^Zabludowicz Center for Autoimmune Diseases, Sheba Medical Center, Sackler Faculty of Medicine, Tel Aviv University, 52621 Tel-Hashomer, Israel

Antiphospholipid syndrome is an acquired thrombophilia characterized by venous or artery thrombosis sometimes accompanied by thrombocytopenia in the presence of antiphospholipid antibodies. The 14th International Antiphospholipid Antibodies Congress was combined with the 4th Latin American Congress on Autoimmunity and took place in Rio de Janeiro in September 2013. In this special issue we have invited several papers that address this modern issue. In this regard, various clinical and molecular aspects such as neurological, cardiovascular, and neonatal antiphospholipid syndrome and also original studies are also presented in this collection as follows.

The article “*Renal transplantation dramatically reduces IgA anti-beta-2-glycoprotein I antibodies in patients with endstage renal disease*” of this issue addresses the frequency of IgA anti-beta2-glycoprotein I positivity in endstage renal disease. The authors found an interesting and significant reduction of these antiphospholipid antibodies after renal transplantations possibly secondary to immunosuppressive therapy.

Another study reviewed the neurological aspects of antiphospholipid syndrome: “*Revisiting the molecular mechanism of neurological manifestations in antiphospholipid syndrome: beyond vascular damage.*” In this article, the authors performed an elegant review on the clinical and mainly on molecular features of thrombotic and nonthrombotic findings of the neurological manifestations of APS. Stroke and transient ischemic attacks are discussed in that paper and also headache, transverse myelitis, cognitive dysfunction, and others as nonthrombotic manifestations of APS.

A review of all cases published in the literature regarding neonatal antiphospholipid syndrome is reported in “*Clinical, laboratory, and therapeutic analyses of 21 patients with neonatal thrombosis and antiphospholipid antibodies: a literature review.*” In this regard, the authors evaluated all clinical aspects of 21 neonatal patients who had the rare neonatal APS manifestation; however, 82% had good outcome.

Rheumatic fever has common clinical and molecular aspects with APS. In this regard, the study “*Rheumatic fever associated with antiphospholipid syndrome: systematic review*” studied all 11 published cases in the world with this rare association. Summarizing, this study demonstrated that common clinical manifestations of APS and rheumatic fever were distributed as follows: carditis with valvular disease in 100% of cases, chorea in two-thirds of the patients, and arthritis in about one-fifth of the cases.

Cardiovascular events are a prominent complication in APS. In this regard, the article “*Cardiovascular risk factors in the antiphospholipid syndrome*” reviews the traditional risk factors and also homocysteine, metabolic syndrome, oxidized LDL, and lipoprotein(a) as novel risk factors for cardiovascular diseases, specifically the role of all these markers in antiphospholipid syndrome.

In an article of this collection, “*Optimization of unnicked *β*2-glycoprotein I and high avidity anti-*β*2-glycoprotein I antibodies isolation,*” the authors modified the in-house isolation and purification procedures of unnicked *β*2-glycoprotein I and high affinity anti-*β*2GPI, improving the purity of antigen and antibodies as well as increasing the number of tests routinely performed with the in-house ELISA.

We hope that this issue will bring you some new data on this important disease and will review concepts that are fundamental for clinical practice.

